# Neural fingerprint of the dark triad: Resting state BOLD power (fALFF) alterations in executive and default mode networks

**DOI:** 10.3758/s13415-025-01352-7

**Published:** 2025-11-04

**Authors:** Richard Bakiaj, Clara Isabel Pantoja Muñoz, Alessandro Grecucci

**Affiliations:** 1 Department of Psychology and Cognitive Sciences (DiPSCo), University of Trento, Corso Bettini, 84, 38068 Rovereto, Italy; 2 Center for Medical Sciences, CISMed, University of Trento, Trento, Italy

**Keywords:** Dark Triad, Unsupervised machine learning, Resting-state fractional amplitude of low-frequency fluctuations, Low-frequency BOLD power, Personality traits

## Abstract

The neural foundations of the Dark Triad (DT) personality traits—narcissism, Machiavellianism, and psychopathy—remain largely unexplored despite their profound impact on social behavior. Previous research has indicated potential structural and functional alterations in the default mode network (DMN) and reward-related regions in individuals exhibiting high levels of DT traits. However, these studies have often been limited by univariate analytical methods and small, unbalanced sample sizes. To address these gaps, we analyzed brain scans from a robust sample of 200 participants (mean age 32.43 years; 105 females) using group Independent Component Analysis (gICA), an unsupervised machine learning approach. This method allowed us to extract blood-oxygen-level-dependent (BOLD) low-frequency spectral power (fractional Amplitude of Low-Frequency Fluctuations; fALFF) associated with resting-state macro-networks without relying on predefined regions of interest. Our analysis identified 20 macro-networks, of which two were significantly predictive of DT traits: the Central Executive Network (CEN) and the posterior hub of the Default Mode Network (DMN). Notably, higher DT scores correlated with increased fALFF within the CEN and decreased fALFF within the DMN. The heightened activity in the CEN may reflect enhanced manipulative abilities and strategic planning commonly observed in individuals with high DT traits, which is supported by the association between the CEN and the Machiavellianism subscale. Conversely, reduced functionality in the DMN may correspond to diminished self-reflective and emotional capacities in these individuals. These findings contribute to a deeper understanding of the neurobiological basis of DT traits and hold implications for future research and interventions.

**Supplementary Information:**

The online version contains supplementary material available at 10.3758/s13415-025-01352-7.

## Introduction

The Dark Triad (DT) describes three partly overlapping but separable “dark” personality traits—narcissism, Machiavellianism, and psychopathy—first articulated by Paulhus and Williams (2002). Elevated DT scores predict a host of costly outcomes, including prejudice, aggression, and broader psychosocial or financial harm (Campbell et al., 2005; Hodson et al., 2009; Kiehl & Hoffman, 2011; Zhu et al., 2021), and they are observed across cultures, indicating a cross-cultural personality dimension (Rogoza et al., 2021).

Briefly, narcissism blends grandiose entitlement with underlying vulnerability (Cain et al., 2008; Pincus et al., 2014), Machiavellianism reflects cold, strategic manipulation and low empathy (Ali et al., 2009; Brewer & Abell, 2017), and psychopathy combines affective callousness with impulsive-antisocial behaviour (Glenn & Raine, 2014; Hare, 2003). Contemporary models, such as the Triarchic Model, view psychopathy along a spectrum from subclinical to clinical levels, focusing on dimensions, such as boldness, meanness, and disinhibition (Patrick et al., 2009). As clinical science increasingly adopts a dimensional approach to personality disorders in general (APA, 2013), understanding subclinical traits is essential for advancing research on personality pathology and refining the conceptualization of personality traits.

Previous studies have mainly examined the subcomponents of the DT separately. For narcissism, alterations in the anterior insula appear to play a significant role, particularly because this region is crucial for empathy, which is often dysfunctional in narcissistic individuals (Decety & Lamm, 2006; Singer & Lamm, 2009). Studies have shown that reduced deactivation in the right anterior insula correlates with higher levels of narcissism during empathy-related tasks (Fan et al., 2011). Structural differences were also observed, such as decreased gray matter volume in frontal-paralimbic regions of those with narcissistic personality traits (Schulze et al., 2013). More recently, Jornkokgoud et al. (2023, 2024) used machine learning to identify a neural circuit involving areas, such as the lateral and middle frontal gyri, angular gyrus, Rolandic operculum, and Heschl’s gyrus, which effectively predicted narcissistic characteristics.

Regarding Machiavellianism, a study by Verbeke et al. (2011) found that individuals high in this trait displayed significant differences in brain regions, such as the basal ganglia, prefrontal cortex (PFC), insula, right hippocampus, and left parahippocampal gyrus, compared with those low in Machiavellianism. Nestor et al. (2013) discovered that people with higher scores on the Machiavellianism-IV scale (MACH-IV) had greater gray matter volume in the left lateral orbital gyrus, which may reflect heightened social manipulation abilities.

In terms of psychopathy, structural brain abnormalities have been consistently found. Psychopaths exhibit reductions in gray matter volume and cortical thinning in regions, such as the dorsolateral prefrontal cortex (dlPFC), orbitofrontal cortex (OFC), and superior temporal gyrus. Additionally, subcortical areas, including the amygdala, hippocampus, and posterior cingulate cortex, also show abnormalities (Yang et al., 2005, 2009a, 2009b). Some studies have found that psychopaths have an increased volume in the striatum, which is linked to reward processing and impulsivity (Choy et al., 2022). Functional connectivity studies have shown that psychopathic individuals display abnormal activity in the Default Mode Network (DMN), which may underlie deficits in self-reflection and moral reasoning (Freeman et al., 2015; Juarez et al., 2012; Pujol et al., 2012).

Research investigating the neurobiological correlates of the DT as a collective construct remains limited, with most studies examining the traits individually. To date, only two studies have explored the neuroanatomical bases of the DT, and none have investigated its functional aspects. Using voxel-based morphometry (VBM), Myznikov et al. (2024) reported lower gray matter volume (GMV) in cingulate, dlPFC and ventral-striatal areas in higher DT scorers. Our own structural/diffusion work (Bakiaj et al., 2025) used data-fusion ML to reveal four DT-relevant networks—reward, executive, DMN and visual—highlighting distributed alterations beyond single voxels.

Taken together, the foregoing studies suggest partial convergence across DT facets. Structural and functional changes in the frontoparietal control system (CEN), limbic‐striatal reward circuitry, and midline DMN hubs are reported across narcissism, Machiavellianism and psychopathy. Differences emerge chiefly in the relative balance of these systems—e.g., narcissism shows stronger midline-DMN involvement, whereas psychopathy shows greater paralimbic and reward anomalies. This pattern guides our expectation that overlapping large-scale networks, rather than entirely facet-specific foci, would predict global DT scores.

Building on the call for revisions to the current Diagnostic and Statistical Manual of Mental Disorders (DSM-5) to include biological markers in diagnostic protocols (APA, 2013; 2017), this study aims to extend our previous work (Bakiaj et al., 2025) by exploring the neural foundations of the DT personality at a functional level. That investigation used only the structural and diffusion volumes; here, with the same dataset, we analyse the resting-state functional magnetic resonance imaging (fMRI) from the same cohort to test whether functional network dynamics echo the structural signatures previously observed. Functional magnetic resonance imaging studies have revealed that personality traits related to the DT influence the activity and connectivity of various brain regions involved in emotional regulation, reward processing, and social cognition, such as the amygdala, prefrontal cortex, and striatum (Carré et al., 2013; Chen et al., 2022; Hevia-Orozco et al., 2022; Li et al., 2017). For instance, Adelstein et al. (2011) examined the relationship between the five-factor personality domains and brain activity, finding unique resting-state functional connectivity patterns for each domain. Neuroticism and Extraversion were notably linked to connectivity in areas involved in emotional regulation and reward processing.

In the current research, we utilized group Independent Component Analysis (gICA; Beckmann et al., 2009; Calhoun et al., 2001), a multivariate, data-driven approach for analyzing the entire brain to detect changes in blood-oxygen-level-dependent (BOLD) signal activity across brain regions. ICA is a method for blind source separation that employs an unsupervised machine learning strategy to uncover distinct, non-overlapping independent neural networks (Bijsterbosch et al., 2017). These independent components represent meaningful, naturally distinct networks that transcend anatomically defined areas, reducing the complexity of brain data into more manageable dimensions (Grecucci et al., 2022a, 2022b). Unlike seed-based correlation methods in resting-state functional connectivity, which depend heavily on seed selection and are prone to bias (Ghomroudi et al., 2024), ICA offers a model-free, whole-brain approach. It separates the brain's BOLD fMRI signal into spatially and temporally independent components, providing a data-driven measure of whole-brain BOLD signals decomposed into spatially independent resting-state networks and their time courses (Ghomroudi et al., 2024). Previous studies have successfully used BOLD signals to identify functional connectivity within and between limbic and prefrontal systems in relation to personality traits (Feng et al., 2018; Vakorin et al., 2011), and in relation to emotional intelligence (Zanella et al., 2022) and anger (Sorella et al., 2021). Low-frequency spectral power of spontaneous BOLD activity—operationalised here as fractional amplitude of low-frequency fluctuations (fALFF)—indexes intrinsic neural excitability and regional arousal (Zou et al., 2008; 2010). We focus on BOLD power (fALFF) rather than coupling-based metrics because the amplitude of spontaneous low-frequency fluctuations provides a region-centric index of intrinsic neural excitability/tonic arousal and shows good test–retest reliability (Zou et al., 2008; 2010). Individual-differences work further links fALFF to behavioral traits relevant to the Dark Triad, including impulsivity and affective instability (Gentili et al., 2020; Xue et al., 2023). Methodologically, fALFF does not require a priori specification of edges or seeds and is thus complementary to seed-based and graph-theoretic connectivity. Combined with gICA, it yields data-driven components and their regional power without seed-selection bias (Beckmann et al., 2009; Bijsterbosch et al., 2017; Calhoun et al., 2001). Because Dark-Triad traits entail altered reward sensitivity and behavioural flexibility, we selected resting-state fALFF (0.008–0.09 Hz) as our principal neural metric.

We hypothesized that the same brain circuits identified in our previous work would be associated with DT traits: A circuit overlapping with the reward network, including regions such as the anterior cingulate cortex, OFC, basal ganglia, amygdala, hippocampus, and thalamus (Haber & Knutson, 2010). A circuit overlapping with the executive network, primarily involving the dlPFC and the lateral posterior parietal cortex. A circuit overlapping with the DMN, including the medial prefrontal cortex, posterior cingulate cortex, precuneus, and angular gyrus (Raichle et al., 2001).

These circuits are relevant to DT traits due to their associated functions in reward processing, executive control, and introspective thinking—areas often found deficient in individuals exhibiting these personality traits. Furthermore, we conducted an exploratory analysis to examine whether these networks could be linked to specific individual traits of the DT (psychopathy, narcissism, and Machiavellianism).

## Materials and methods

### Participants

This study involved a selection of 214 native German speakers drawn from the "MPI-Leipzig MindBrain-Body" database (available on OpenNeuro: https://openneuro.org, dataset number ds000221; referenced from Babayan et al., 2020; Harvard Dataverse, 2020). This database comprises structural and functional MRI data, along with behavioral assessments, from a total of 321 German-speaking individuals. Participants were included based on specific criteria outlined below, among which they had completed the Short Dark Triad (SD3) questionnaire, were suitable for MRI scanning, and had no history or current diagnosis of psychiatric or neurological disorders. To minimize potential confounding effects associated with aging, we excluded 17 participants who were 70 years or older. Additionally, one participant was excluded during data analysis. The final sample consisted of 200 participants (96 females), ranging in age from 20 to 69 years (mean age = 32.37 years, standard deviation = 13.9). Available demographics were age and sex. Socioeconomic status and race/ethnicity were not available in the publicly released dataset and therefore could not be analysed.

### Exclusion criteria

Exclusion criteria included: history of psychiatric diseases that required inpatient treatment for longer than 2 weeks within the last 10 years (e.g., psychosis, attempted suicide, posttraumatic stress disorder); history of neurological disorders (including multiple sclerosis, stroke, epilepsy, brain tumour, meningoencephalitis, severe concussion); history of malignant diseases; intake of one of the following medications, any centrally active drugs (including Hypericum perforatum); positive drug anamnesis (extensive alcohol, MDMA, amphetamines, cocaine, opiates, benzodiazepine, cannabis). Specific MRI exclusion criteria included any metallic implants, braces, non-removable piercings; tattoos; pregnancy; claustrophobia; tinnitus; surgical operation in the last 3 months. Written informed consents were obtained by all participants, who received financial compensations for their participation. The study protocol was approved by the ethics committee of the University of Leipzig (097/15-ff) (154/13-ff) (Babayan et al., 2019; Mendes et al., 2019).

### Short dark triad (SD3) questionnaire

The SD3 questionnaire is an integrated tool designed to assess the three DT traits—Machiavellianism, narcissism, and psychopathy—by combining elements from established scales: MACH-IV, the Narcissistic Personality Inventory (NPI), and the Self-Report Psychopathy Scale (SRP-III) (Jones & Paulhus, 2014; Paulhus & Jones, 2015). The SD3 comprises 27 items, with nine items dedicated to each sub-trait, and responses are recorded on a 5-point Likert scale ranging from 1 ("strongly disagree") to 5 ("strongly agree"). Example items include: "There are things you should hide from other people because they don’t need to know" (Machiavellianism); "I have been compared to famous people" (narcissism); and "Payback needs to be quick and nasty" (psychopathy). For the purpose of this study, the SD3 was translated into German.

Descriptive statistics for each dimension of the SD3 are detailed in Table 1 of the supplementary material, including means, standard deviations, standard errors, minimums, maximums, ranges, and 95% confidence intervals. According to normative data (Table 2 of the supplementary material) proposed by Paulhus (available at https://www.psytoolkit.org/survey-library/short-dark-triad.html), which have been adjusted for the nine-item subscales, our sample's mean scores fall within the normal range. Specifically, the mean scores were 20.74 for Machiavellianism, 24.50 for narcissism, and 18.66 for psychopathy. The adjusted normal ranges are mean scores of 27.9 for Machiavellianism, 25.2 for narcissism, and 21.6 for psychopathy, with thresholds for elevated scores at 34.74, 33.12, and 30.6, respectively. These findings suggest that the participants in our study exhibited DT traits within normative ranges typical of the general population.

The reliability of the SD3 in this sample was assessed using Cronbach's alpha coefficients, which indicated acceptable internal consistency: 0.68 for Machiavellianism (compared with 0.78 in the original English version), 0.65 for narcissism (original 0.77), and 0.59 for psychopathy (original 0.8). Total SD3 scores were calculated by summing the individual scores for each of the three DT traits. Because item-level responses were not available in the open-source dataset, we could not compute Cronbach’s α for the overall Dark-Triad score. The three SD3 subscales showed positive but modest intercorrelations—Machiavellianism–Narcissism *r* =.17 (*p* =.018), Machiavellianism–Psychopathy *r* =.31 (*p* <.001), Narcissism–Psychopathy *r* =.32 (*p* <.001)—consistent with partial overlap among facets. Accordingly, we use the SD3 total as an index of shared ‘dark’ variance, while also inspecting facet-specific associations (Paulhus & Williams, 2002; Table 3 of the supplementary material).

### MRI data acquisition

High-resolution T1-weighted images were obtained using a 3 Tesla Siemens MAGNETOM Verio scanner (Siemens Healthcare GmbH, Erlangen, Germany) equipped with a 32-channel head coil. The data collection was conducted at the Day Clinic for Cognitive Neurology of the University Clinic Leipzig and the Max Planck Institute for Human Cognitive and Brain Sciences (MPI CBS) in Leipzig, Germany. While the original MPI-Leipzig Mind-Brain-Body dataset includes structural magnetic resonance imaging, fMRI, and diffusion-weighted imaging scans (Babayan et al., 2019), our study focused exclusively on the T1-weighted images. The structural images were acquired using a three-dimensional Magnetization Prepared 2 Rapid Acquisition Gradient Echoes (3D MP2RAGE) sequence (Marques et al., 2010) with the following parameters: sagittal acquisition orientation; one 3D volume consisting of 176 slices; repetition time (TR) = 5,000 ms; echo time (TE) = 2.92 ms; inversion times (TI1) = 700 ms and (TI2) = 2,500 ms; flip angles (FA1) = 4°, (FA2) = 5°; prescan normalization applied; echo spacing = 6.9 ms; bandwidth = 240 Hz/pixel; field of view (FOV) = 256 mm; isotropic voxel size of 1 mm; GRAPPA acceleration factor of 3; interleaved slice order; and a total scan duration of 8 min and 22 s. In addition to the structural imaging, standard T2-weighted imaging was performed to complement the analysis. Functional imaging data were acquired using a T2*-weighted gradient-echo echo-planar imaging (EPI) multiband sequence for BOLD resting-state fMRI (rs-fMRI). During the rs-fMRI scan, participants were instructed to remain awake, minimize movement, and keep their eyes open while fixating on a low-contrast crosshair. This setup aimed to capture authentic resting-state neural activity without interference from sleep or engagement in specific cognitive tasks. Although explicit monitoring of participants' wakefulness was not conducted during the rs-fMRI scan, it was presumed that all participants adhered to the instructions to stay awake throughout the session. The rs-fMRI scanning protocol was precisely defined with a repetition time (TR) of 1,400 ms and a total of 657 volumes collected, providing an extensive dataset for analyzing temporal variations in brain activity (Babayan et al., 2019).

### Pre-processing

Functional MRI data were preprocessed using the CONN toolbox (Nieto-Castanon, 2020), a comprehensive software suite for functional connectivity analysis. We employed the toolbox's default preprocessing pipeline to prepare the fMRI data for subsequent analysis, a critical step in minimizing noise and artifacts and enhancing the interpretability of the results. The preprocessing steps included functional realignment to correct for head motion by aligning all functional images to a reference image, accounting for any movement during scanning. Slice-timing correction was applied to adjust for temporal differences in slice acquisition times within each volume, synchronizing the timing of the voxel time series. The functional images were then coregistered with each participant's structural T1-weighted image to ensure anatomical correspondence between functional and structural data. Subsequently, the structural T1 images were segmented into different tissue types—GM, WM, and cerebrospinal fluid—to improve normalization accuracy and allow for tissue-specific analyses. Normalization involved applying nonlinear transformations to warp individual brains into a standard anatomical space, specifically the Montreal Neurological Institute template, facilitating group-level comparisons. Spatial smoothing was applied to the functional data using a Gaussian kernel of 8 mm full-width at half maximum, which enhances the signal-to-noise ratio and compensates for inter-subject anatomical variability. Artifact and outlier detection was conducted to identify and mitigate the effects of motion-related artifacts and other anomalies, thereby reducing physiological noise and other confounding factors. Additionally, band-pass filtering and denoising procedures were implemented to minimize physiological and non-neuronal sources of noise. This was achieved through regression of nuisance variables and may have included data-driven noise reduction methods to further enhance data quality. Denoising followed CONN’s default aCompCor pipeline: five PCA components from WM and five from CSF, the 6 rigid-body motion parameters + first derivatives, linear trends, and outlier frames identified by ART (framewise displacement > 0.5 mm or global signal Z > 3). fALFF was computed from residual time-series by fast Fourier transform (FFT), estimating band-limited power (0.008–0.09 Hz) and dividing by total power (0–0.25 Hz), following standard definitions (Zou et al., 2008; 2010).

### Group-Independent Component Analysis (gICA) for networks decomposition

We employed an unsupervised machine learning approach, specifically Independent Component Analysis (ICA; Xu et al., 2009), to analyze the functional imaging data. Group-Independent Component Analysis (gICA), performing ICA on the group data by temporal concatenation (Beckmann et al., 2009; Calhoun et al., 2001), offers several advantages for resting-state analyses, notably its effectiveness in identifying and removing noise from the data and enhancing the statistical independence of datasets. This facilitates the extraction of multiple consistent networks (Rajamanickam, 2020). The primary rationale for adopting ICA was to delineate resting-state networks by leveraging the unique characteristics of the participants' data without imposing pre-established criteria. This data-driven approach is particularly advantageous for discerning organically formed patterns of functional connectivity across brain regions, which are not confined to the predetermined boundaries typically associated with network nodes. Consequently, it enables a more individualized and nuanced understanding of brain connectivity (Kornelsen et al., 2020; Motoyama et al., 2019). Considering that resting-state networks can vary significantly between individuals, accounting for personal differences is crucial in the analysis. Following the preprocessing phase and careful quality assurance checks using diagnostic plots, we proceeded with ICA without employing predetermined seeds or regions of interest (ROI). This decision aimed to avoid limiting the analyses and to explore potential differences in connectivity across the entire brain that might be associated with DT traits.

Group Independent Component Analysis (gICA) is employed in this study as an unsupervised machine learning technique because it operates on a dataset without being given predefined labels or target variables (Vieira et al., 2020). Unlike supervised methods, which learn mappings between inputs and known outputs, unsupervised approaches like gICA aim to uncover latent structures or patterns within the data. Specifically, gICA identifies statistically independent spatial components based on similarities in functional activation across participants, enabling the extraction of meaningful brain networks without prior assumptions. A key advantage of gICA over traditional single-subject ICA is its ability to extend the analysis to multi-subject datasets by estimating group-level components that are common across individuals, while also preserving subject-specific variability (Calhoun et al., 2001; Beckmann et al., 2009). This makes it particularly suitable for identifying intrinsic connectivity networks that may underlie shared neural processes or dysfunctions in clinical populations.

**Fig. 1:**
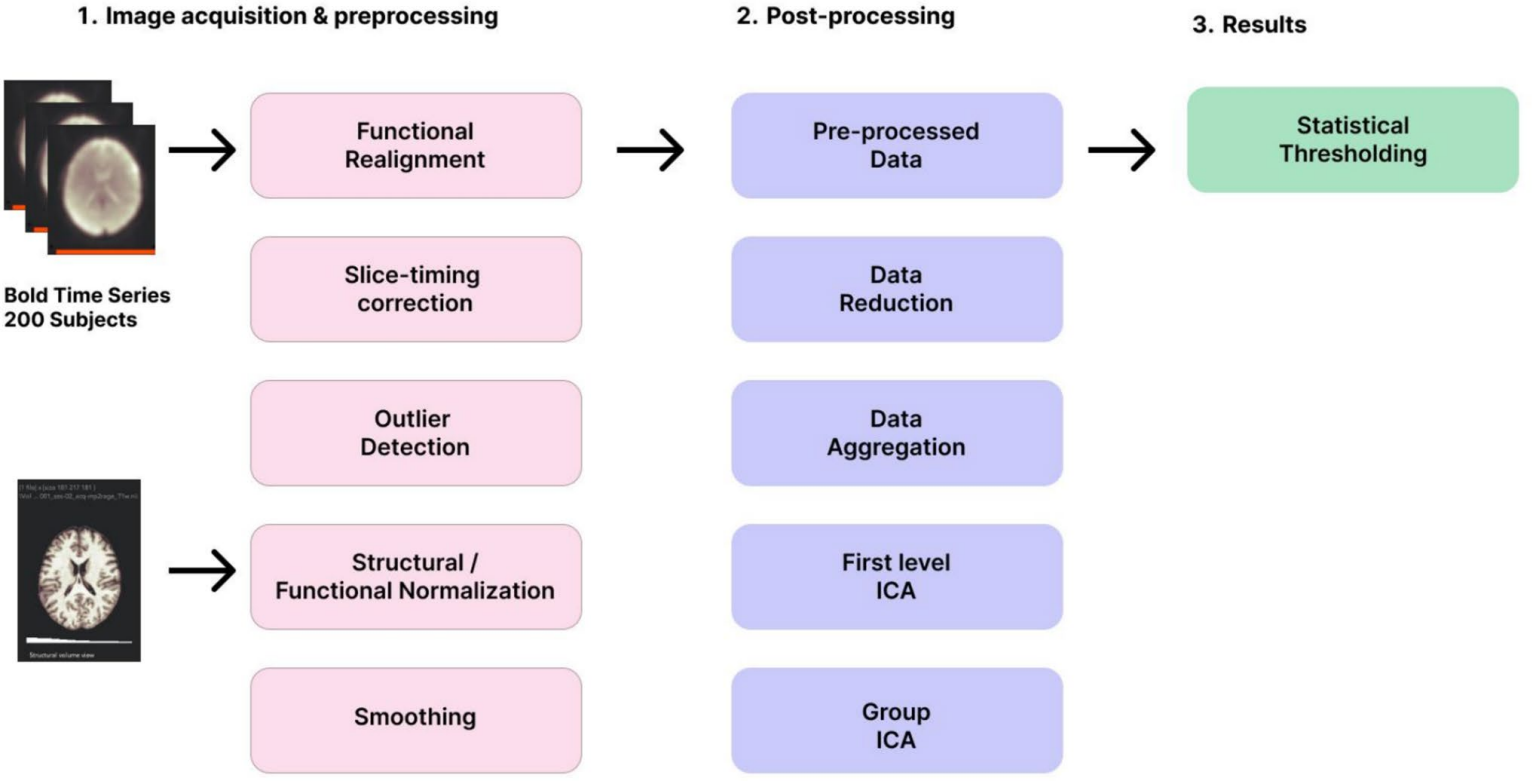
Methodology. First, the resting state data were preprocessed. Then, 20 independent components were extracted by using an unsupervised machine learning Group ICA approach and fALFF extraction

We selected a voxel-to-voxel analysis approach, specifying group-level ICA. Using default settings, the ICA identified 20 networks. The analysis followed the methodology of Calhoun and colleagues (2001) for group-level ICA, encompassing several critical steps: preconditioning through variance normalization, concatenation of subjects' BOLD signal data across the temporal dimension, dimensionality reduction at the group level, application of FastICA to estimate independent spatial components, and the use of GICA1 back-reconstruction to generate individual subject spatial maps (Nieto-Castanon, 2020). Figure 1 provides an illustrative overview of the methodology employed to analyze the participants' brain networks and predict their personality traits.

### Stepwise regression and correlation analyses

After performing brain decomposition using ICA, we focused on the low-frequency spectral-power coefficients (fALFF) of each network for each participant. These coefficients were utilized in a linear regression model with backward selection procedure to predict DT scores based on the brain networks identified by group ICA. A backward stepwise regression analysis was conducted using JASP software (JASP Team, Version 0.16.2, 2022). Initially, all predictors were entered into the model simultaneously. Predictors were then sequentially removed based on established statistical criteria: inclusion of predictors was set at a significance level of *p* <.05, while exclusion was defined by a threshold of *p* >.01. All regression models included age and sex as covariates alongside the neural fALFF predictors. As a sensitivity analysis, we re-estimated the final regression omitting sex and age to assess whether brain–DT associations were contingent on sex and age differences. Subsequently, Pearson's correlation tests were applied to identify potential associations between the significant networks and the DT subscale scores (Machiavellianism, Narcissism, and Psychopathy). To analyze gender effects on these relationships, independent samples *t*-tests were conducted.

## Results

Resting-state network decomposition was performed using a data-driven gICA implemented in CONN, which identified 20 independent components (IC). To distinguish noise components from intrinsic resting-state networks, each IC was visually inspected and compared against CONN's network atlas using the spatial match-to-template function. Subsequently, for every IC we extracted fractional amplitude of low-frequency fluctuations (fALFF; 0.008–0.09 Hz power/total power) using CONN’s ROI Frequency module. To control for Type I errors, a cluster-size-based false discovery rate (FDR) correction was applied (p < 0.05), with voxel-level thresholding at *p* <.001 within each analysis.

### Macro network contributions to overall dark triad

**Fig. 2:**
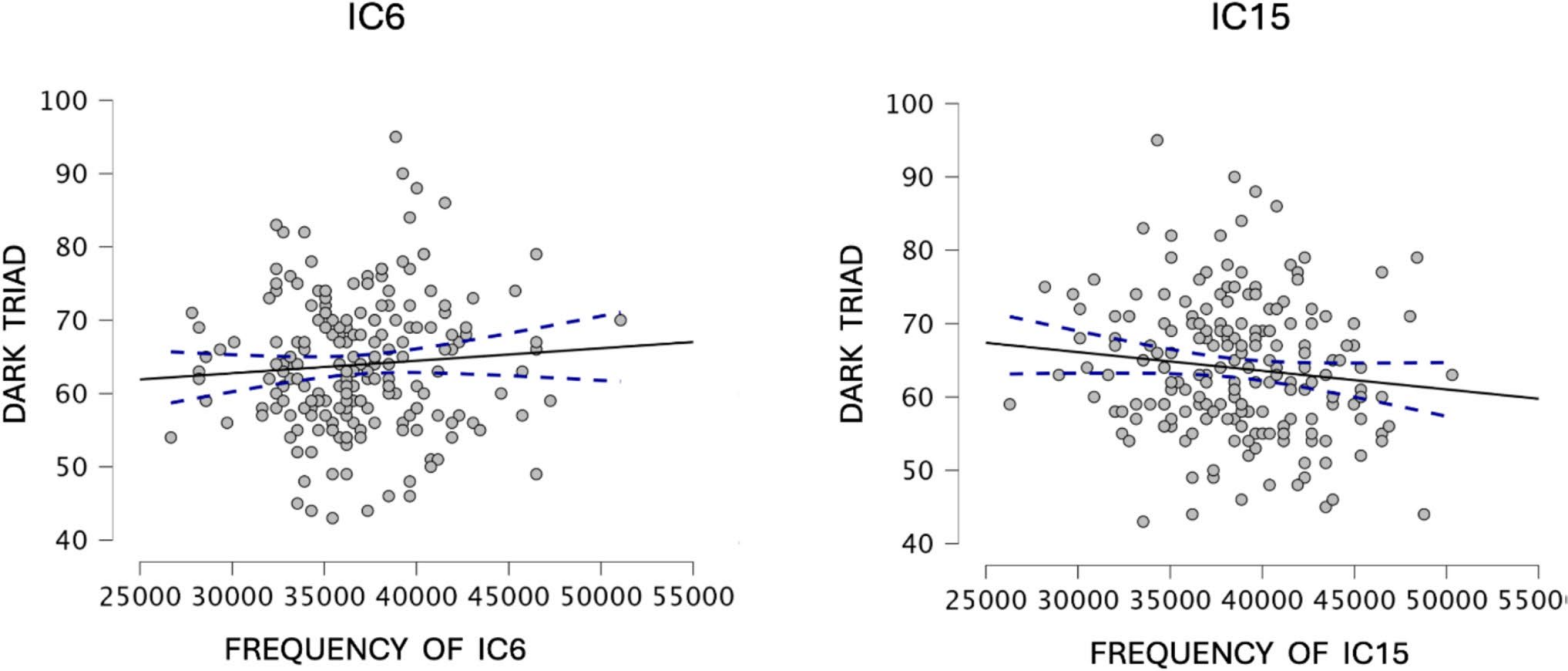
Brain–behaviour associations retained by the backward-stepwise regression. **Left:** The left plot represents the positive relation between the DT total score and the fALFF in IC6, corresponding to the CEN. **Right:** The right plot represents the negative relation between the DT and the fALFF in IC15, corresponding to the posterior DMN hub. Each point represents one participant; the best-fitting regression line is overlaid. DT = Dark Triad; fALFF = fractional amplitude of low-frequency fluctuations; CEN = Central Executive Network; DMN = Default-Mode Network; IC = Independent Component

**Fig. 3:**
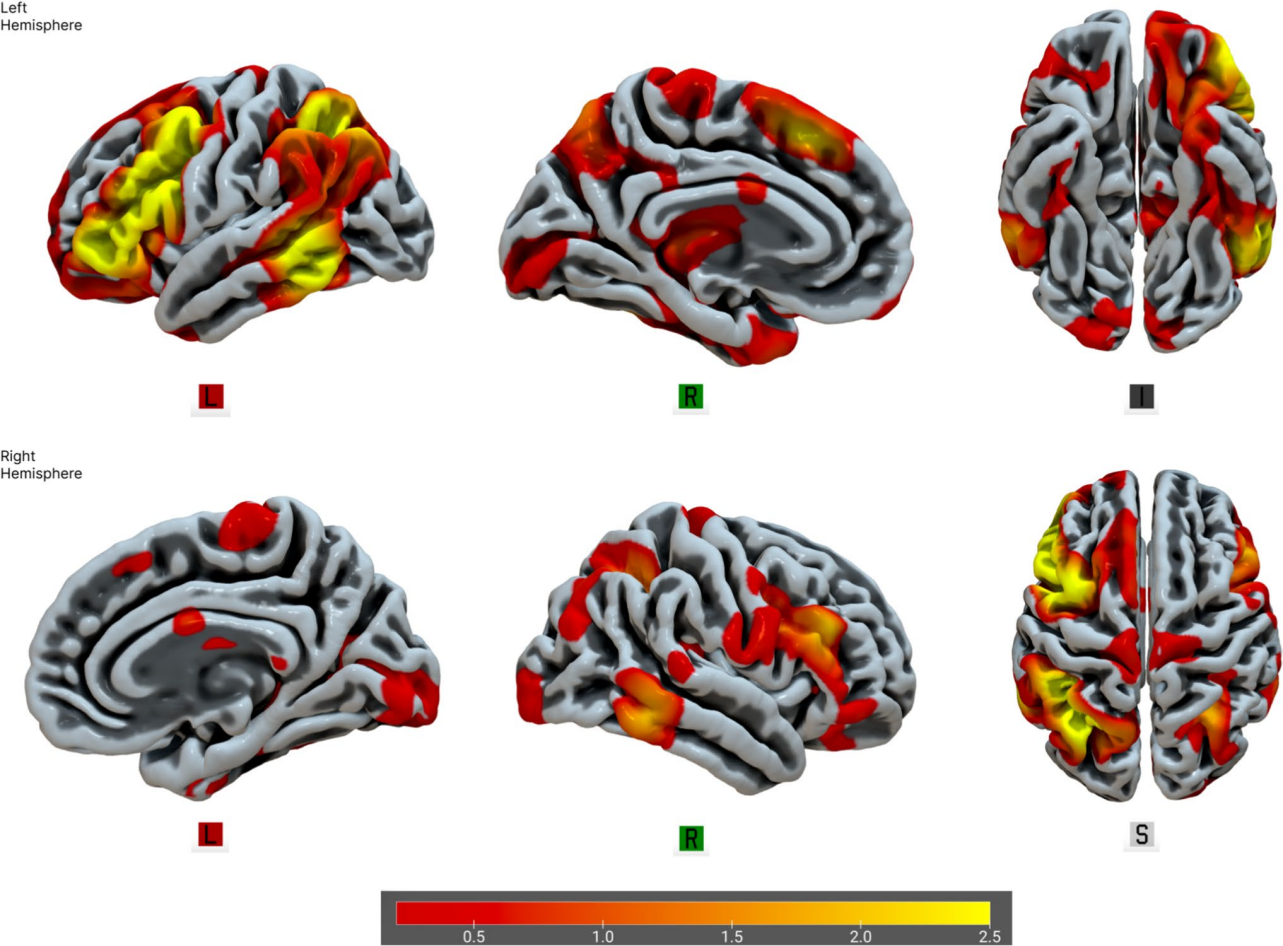
IC6, The Central Executive Network. From left to right are displayed brain plots of IC6. Regions with increased values are represented with warm colors. IC6 showed an increase in fALFF in higher DT individuals

**Fig. 4:**
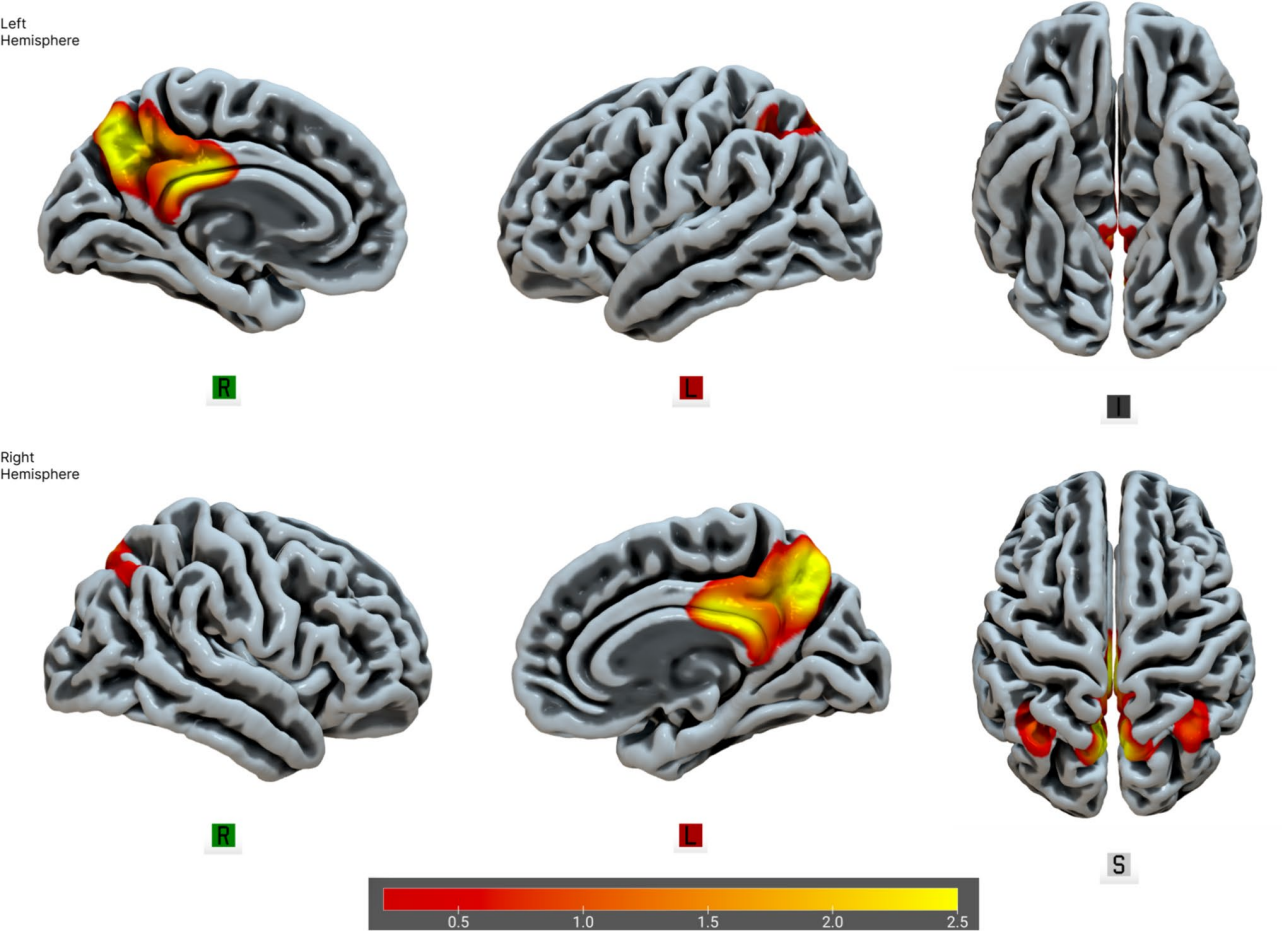
IC15—the posterior hub of the DMN. From left to right are displayed brain plots of IC15. IC15 showed a decrease in fALFF in higher DT individuals

The backward multiple regression analysis (including sex and age as covariates) yielded a significant model (F(4,195) = 6.029, *p* <.001, R^2^ =.084, adj. R^2^ =.07), indicating that the fALFF of two networks, IC6 (unstandardized 3.934 × 10⁻^4^, SE 1.674 × 10⁻^4^, standardized β =.173, *t* = 2.350, *p* =.02) and IC15 (unstandardized − 3.525 × 10⁻^4^, SE 1.563 × 10⁻^4^, standardized β =  −.163, *t* =  − 2.255, *p* =.025), predicted overall DT scores. Sex was also a significant covariate (unstandardized − 4.424, SE 1.274, standardized β =  −.242, *t* =  − 3.473, *p* <.001), consistent with higher DT scores in men than women in this sample. These networks correspond to well-established resting-state networks, specifically the CEN, and the posterior hub of the DMN. The higher the DT trait, the higher the CEN and the lower the DMN fALFF. Please refer to Figs. 2, 3 and 4 for visual representations of the network areas (see Tables 4 and 5 for the anatomical regions of interest in the supplementary material). Multicollinearity was negligible, with a modest inter-correlation (*r* =.34) and a variance-inflation factor of VIF = 1.13, well below the conservative cutoff of 5 recommended for neuroimaging regression (O’Brien, 2007). Note that the IC6–IC15 correlation (*r* =.34; VIF = 1.13) pertains to collinearity diagnostics in a regression context and indicates negligible multicollinearity; this statistical consideration is distinct from the substantive (and modest) overlap among DT subscales (*r* =.17–.32).

We also conducted a sensitivity analysis, omitting sex and age. Only without sex, model fit diminished to marginal levels, F(3,196) = 2.88, *p* =.058, R^2^ =.028, adj. R^2^ =.019; IC6 (standardized β =.147, *p* =.06) and IC15 (standardized β =  −.12, *p* =.125) no longer reached conventional significance. This pattern underscores the importance of modelling sex; all primary inferences are based on the sex- and age-adjusted model.

### Additional analyses

Subsequently, we conducted correlation analyses to evaluate the relationships between the significant networks (IC6 and IC15) and the individual DT subtraits. For the distribution of DT traits and the rationale behind the choice of statistical tests based on these distributions, see Figs. 5 and 6 in the supplementary material. The analysis revealed a significant positive correlation between IC6 and the Machiavellianism subscale (Spearman's ρ = 0.162, *p* < 0.023), suggesting that increased fALFF in IC6 is associated with higher Machiavellianism scores.

### Relationship between sex, age, and dark-triad traits

The participants were approximately equally distributed by sex, with 96 females and 104 males, and ranged in age from 20 to 70 years. The average overall DT total score on the SD3 questionnaire was 63.90 (standard deviations [*SD*] = 9.16) out of a maximum of 135, with mean subscale scores of 20.74 (*SD* = 3.8) for Machiavellianism, 24.5 (*SD* = 4.72) for narcissism, and 18.66 (*SD* = 4.27) for psychopathy.

Separate analyses by sex indicated that males had a higher DT total score (*M* = 65.73, *SD* = 9.2) compared with females (*M* = 61.89, *SD* = 8.73). A two-sample *t*-test assuming unequal variances revealed that this difference was statistically significant (t(199) =  − 3.04, *p* = 0.001, one-tailed). Further analysis showed that males (*M* = 19.75, *SD* = 4.16) also scored significantly higher than females (*M* = 17.46, *SD* = 4.08) on the psychopathy subscale (t(199) = 3.94, *p* < 0.001, one-tailed).

Regarding the effect of age on the DT, Spearman's correlation revealed no significant associations between age and Machiavellianism (ρ = 0.041, *p* = 0.142), age and narcissism (ρ =  − 0.062, *p* = 0.384), age and psychopathy (ρ =  − 0.120, *p* = 0.09), or age and the DT total score (ρ =  − 0.045, *p* = 0.53).

## Discussion

This study aimed to identify the neural functional correlates of the DT personality traits—narcissism, Machiavellianism, and psychopathy—using a data-driven gICA approach. Two independent networks, IC6 and IC15, emerged as significant predictors of total DT scores. IC6 overlapped with the CEN, while IC15 corresponded to the posterior hub of the DMN. Notably, higher DT scores were associated with increased fALFF in the CEN (IC6) and decreased fALFF in the DMN (IC15). Furthermore, IC6 was specifically correlated with Machiavellianism. Importantly, the relationship persists after controlling for age, sex and motion, and mirrors structural covariance we previously reported in the same cohort (Bakiaj et al., 2025). Together, these findings point to network-level rather than region-specific alterations as a core neurobiological signature of dark personality features. Given that DT facets correlate only modestly, effects for the DT total should be interpreted as operating on shared variance rather than implying interchangeability of the three traits. The specific link between IC6 and Machiavellianism further suggests facet-level specificity within this shared architecture.

fALFF indexes intrinsic neural excitability and has been linked to cognitive flexibility and affective regulation (Gentili et al., 2020; Xue et al., 2023; Zuo et al., 2010). Higher fALFF in the CEN (IC6) may reflect increased tonic arousal and strategic cognitive control among individuals high in DT traits, whereas reduced fALFF in the posterior DMN (IC15) may indicate diminished self-referential and socio-affective processing. Together these opposing patterns suggest that elevated “dark” personality features are supported by a neural profile of enhanced goal-directed vigilance coupled with dampened introspective activity.

### Role of Central Executive Network (CEN) for dark triad and machiavellianism

Our study found that IC6 exhibited increased fALFF and was associated with the CEN, a neural system that governs goal maintenance and flexible attention (Cooper et al., 2017). Functionally, a higher low-frequency power here may reflect a chronically “primed” state that facilitates strategic monitoring of the social environment—skills that underlie successful manipulation and deception.

Previous research has demonstrated the involvement of the CEN in DT personality traits both collectively and in individual components, at neuroanatomical and cognitive levels. Specifically, our earlier structural work in the same cohort (Bakiaj et al., 2025) pointed to denser grey-matter covariance across identical CEN territories, and voxel-based studies (Myznikov et al., 2024) report similar prefrontal changes. The present results therefore converge on a structure–function motif: more tissue, or more baseline excitability, in executive circuits tracks darker personality tendencies.

Furthermore, individuals with high DT traits (especially psychopaths and Machiavellians) are often adept at deception to manipulate others and achieve their objectives (McDonald et al., 2012). Their tendency to present themselves as friendly and trustworthy may enhance their ability to deceive (Michels et al., 2020; Vrij et al., 2010). Considering that lying is cognitively demanding—requiring multiple cognitive processes beyond simple recall (Vrij et al., 2008; Zuckerman et al., 1981)—the involvement of the CEN may reflect the cognitive demands associated with deceptive behaviors.

We also found a positive association between IC6 and Machiavellianism, suggesting that the enhanced cognitive control functions of the CEN may underpin the strategic thinking and manipulative behaviors characteristic of individuals high in Machiavellian traits. Machiavellians are known for their ability to navigate complex social environments, formulate long-term goals, and evaluate the consequences of their actions (Jones & Paulhus, 2011). These cognitive abilities align with the functions of the CEN, which integrates multiple information streams for adaptive decision-making, problem-solving, and social cognition (Gu et al., 2015).

Our findings are consistent with previous studies linking DT traits and Machiavellianism to increased activity in brain areas involved in strategic thinking and social manipulation. For example, Bereczkei (2015) found that individuals with high Machiavellian traits exhibited increased activity in the inferior frontal gyrus during decision-making tasks involving social interactions. This region, part of the CEN, is crucial for interpreting social cues and predicting others' behavior. Additionally, Gong et al. (2023) identified a significant correlation between Machiavellianism and gray matter volume in the frontal gyrus, further supporting the involvement of this region in manipulative behaviors.

Other brain regions identified in IC6, such as the temporal gyrus and thalamus, have also been implicated in Machiavellianism. The thalamus, a central hub for information processing and sensory integration, plays a role in controlling attention and regulating the flow of information between different brain areas (Barlow et al., 2010). Its involvement in Machiavellianism may reflect enhanced attentional control and the ability to selectively focus on information relevant to achieving strategic goals.

### Default mode network and dark-triad traits

In contrast to IC6, IC15 exhibited decreased fALFF in regions associated with the DMN hub, which is involved in self-referential thinking, social cognition, and emotional regulation (Andrews-Hanna et al., 2010). Dampened spontaneous activity in this self-referential network plausibly translates into blunted introspection and empathy—features common to narcissism, psychopathy, and Machiavellianism (Paulhus & Williams, 2002). The DMN plays a crucial role in theory of mind and mentalizing abilities, which are essential for understanding others' intentions and emotions (Schurz et al., 2021). Reduced DMN activity in individuals with high levels of DT traits may lead to difficulties in forming deep social connections and a lack of consideration for others' feelings.

Research supports the association between DMN dysfunction and traits of psychopathy and narcissism. For instance, Cao et al. (2022) identified abnormalities in DMN functioning among individuals with narcissistic personality disorder, suggesting that disruptions in this network may contribute to impaired self-reflection and inflated self-importance. Similarly, Baskin-Sommers et al. (2014) found that psychopathic traits are linked to diminished DMN activity, which may underlie the emotional detachment and impulsivity often observed in these individuals.

Furthermore, IC15 encompassed areas within the posterior DMN, including the parieto-occipital region, which has been implicated in impulsivity. Lapomarda et al. (2021a, 2021b) reported that decreased activity in this region was associated with increased impulsivity in individuals with bipolar disorder. This finding may be relevant to the impulsive and risk-taking behaviors seen in individuals with high psychopathic and narcissistic traits. Reduced DMN activity may impair their ability to engage in future-oriented thinking, leading to a lack of long-term planning and a propensity for risky behaviors (Fulton et al., 2010; Huang et al., 2019; Snowden et al., 2017).

The DMN's role in emotional regulation is another critical factor. Diminished activity in this network may contribute to the emotional dysregulation observed in psychopathy, characterized by a lack of remorse and emotional detachment (Pan et al., 2018). In narcissism, impaired emotional regulation might manifest as hypersensitivity to criticism and fluctuations in self-esteem, with individuals lacking the introspective capacity to temper their grandiose self-perception (Cain et al., 2008; Jornkokgoud et al., 2023; Pincus et al., 2014).

## Conclusions and limitations

This study offers new insights into the neurobiological underpinnings of DT personality traits by employing a data-driven machine learning approach. Using gICA, we identified distinct resting-state networks associated with DT traits, revealing patterns of intrinsic low-frequency BOLD activity (fALFF) without the constraints of predefined regions of interest. The findings highlight the significant role of the CEN in DT traits overall and Machiavellianism specifically, as well as the involvement of the DMN in shared features of DT traits, such as impaired self-reflection and emotional regulation.

By elucidating the neural correlates associated with DT traits, this study enhances our understanding of how alterations in functional network dynamics contribute to these complex personality dimensions. The use of fALFF as a measure provides a nuanced view of neural activity, emphasizing the importance of functional network complexity and information integration in personality traits. These insights may inform the development of targeted interventions aimed at addressing the cognitive and emotional dysfunctions characteristic of extreme and pathological DT profiles.

While the study presents valuable contributions, certain limitations should be acknowledged. The reliance on a single psychometric measure of DT traits may not fully capture the complexity and nuances of these personality dimensions. Future research should incorporate multiple assessment tools to provide a more comprehensive evaluation of DT traits and to verify the consistency of findings across different measures. In this regard, another thing worth mentioning is that item-level scores from the SD3 were not released with the Mind-Brain-Body dataset, so a reliability coefficient (e.g., Cronbach’s α) for the composite Dark-Triad index could not be computed. Although the three subscales showed the expected positive inter-correlations, future studies that retain item-level data should formally verify the internal consistency of the unified DT construct. Furthermore, socio-economic status (SES) and race/ethnicity were not available in the open-access release of the Mind–Brain–Body dataset, so we could not examine their relation to DT traits. Future work using datasets that include these variables is needed. Additionally, our analysis focused on fALFF as the primary measure of intrinsic regional activity (low-frequency BOLD power). Exploring other connectivity metrics, such as graph theoretical measures or region-to-region (ROI-to-ROI) connectivity analyses, could further enhance our understanding of the functional bases of DT traits. Such approaches may reveal additional aspects of neural network organization and inter-regional communication pertinent to DT characteristics. In addition, although fALFF is relatively insensitive to sampling rate, the 1.4-s TR may attenuate estimates of very-high-frequency variability and should be considered when generalising our findings. Longitudinal studies are also recommended to assess the stability and progression of the observed brain-behavior relationships over time. This would help determine whether the neural patterns identified are consistent traits or if they change in response to interventions or developmental factors. Understanding the temporal dynamics of these neural correlates could provide deeper insights into the potential for modifying pathological DT traits through therapeutic means.

In summary, this research contributes to the growing body of literature on the neural mechanisms underlying DT personality traits by applying a data-driven approach. The identification of specific neural networks associated with DT traits advances our knowledge of the biological foundations of these complex personalities. By integrating neuroimaging techniques with psychological assessments, we move closer to a more holistic understanding of personality disorders, which may ultimately lead to improved diagnostic tools and therapeutic strategies. Continued exploration in this field holds promise for uncovering the intricate interplay between brain function and personality, paving the way for interventions that can enhance psychological well-being.

## Supplementary Information

Below is the link to the electronic supplementary material.

Supplementary file1 (DOCX 246 kb)

## Data Availability

The dataset analysed during the current study is available in the MPI-Leipzig_Mind-Brain-Body repository, https://openneuro.org/datasets/ds000221/versions/1.0.0 (accessed 1 April 2022). The complete LEMON Data can be accessed via Gesellschaft für wissenschaftliche Datenverarbeitung mbH Göttingen (GWDG) https://www.gwdg.de/ (accessed 1 April 2022). Raw and preprocessed data at this location are accessible through web browser https://ftp.gwdg.de/pub/misc/MPI-Leipzig_Mind-Brain-Body-LEMON/MRI_MPILMBB_LEMON/ (accessed on 1 December 2021) and a fast FTP connection [ftp://ftp.gwdg.de/pub/misc/MPI-Leipzig_Mind-Brain-Body-LEMON/ (accessed 1 April 2022)].

## Code availability

Upon reasonable request, software application or custom code would be made available.
