# Supplementary material for: Neural fingerprint of the dark triad: Resting state BOLD power (fALFF) alterations in executive and default mode networks
Source: Cogn Affect Behav Neurosci. 2025 Nov 4;26(1):139–54. doi: 10.3758/s13415-025-01352-7 (PMC12847167; doi:10.3758/s13415-025-01352-7)
Supplement: Supplementary file 1 — Supplementary file1 (DOCX 246 kb) [file 13415_2025_1352_MOESM1_ESM.docx]

**Supplementary Figures and Tables**

**Abbreviations**

DT: Dark Triad

SD3: Short Dark Triad

DD: Dirty Dozen

MACH-IV: Machiavellianism-IV scale

NPI: Narcissistic Personality Inventory

SPR-III: Self-Report Psychopathy Scale

PFC: Prefrontal Cortex

OFC: Orbitofrontal Cortex

GM: Gray Matter

GMV: Gray Matter Volume

WM: White Matter

MRI: Magnetic Resonance Imaging

fMRI: functional Magnetic Resonance Imaging

sMRI: structural Magnetic Resonance Imaging

MP2RAGE: Magnetization Prepared 2 Rapid Acquisition Gradient Echoes

dlPFC: Dorsolateral Prefrontal Cortex

DMN: Default Mode Network

CEN: Central Executive Network

ACC: Anterior Cingulate Cortex

tIVA: transposed Independent Vector Analysis

DTI: Diffusion Tensor Imaging

mPFC: medial Prefrontal Cortex

PCC: Posterior Cingulate Cortex

DWI: Diffusion-Weighted Imaging

sMRI: structural Magnetic Resonance Imaging

MNI: Montreal Neurological Institute

SPM12: Statistical Parametric Mapping

CAT12: Computational Anatomy Toolbox

BSS: Blind Source Separation

ICA: Independent Component Analysis

SD: Standard Deviation

VBM: Voxel-Based Morphometry

DSM-5: Diagnostic and Statistical Manual of Mental Disorders

RSFC: Resting-State Functional Connectivity

gICA: Group Independent Component Analysis

BOLD: Blood-Oxygen-Level-Dependent

EPI: Echo-Planar Imaging

rs-fMRI: Resting-State functional Magnetic Resonance Imaging

TR: Repetition Time

FWHM: Full Width at Half Maximum

ROIs: Regions of Interest

IC: Independent Component

FDR: False Discovery Rate

fALFF: fractional Amplitude of Low-Frequency Fluctuations

**Table 1. Descriptive Statistics of the SD3**

|  | **SD3_Mach** | **SD3_Narc** | **SD3_Psycho** | **SD3_Total** |
| --- | --- | --- | --- | --- |
| **Mean** | 20.74 | 24.5 | 18.66 | 63.9 |
| **Std. Deviation** | 3.81 | 4.72 | 4.27 | 9.16 |
| **Std. Error** | 0.27 | 0.33 | 0.3 | 0.65 |
| **Min** | 11 | 13 | 9 | 43 |
| **Max** | 33 | 41 | 31 | 95 |
| **Range** | 22 | 28 | 22 | 52 |
| **95% CI Lower** | 20.21 | 23.85 | 18.07 | 62.63 |
| **95% CI Upper** | 21.26 | 25.15 | 19.25 | 65.16 |

**Table 2. Normal Ranges for the Dark Triad traits**

| **Trait** | **Mean** | **Standard Deviation** | **Score above this is outside the normal range** |
| --- | --- | --- | --- |
| **Machiavellianism** | 3.1 | 0.76 | > 3.86 |
| **Narcissism** | 2.8 | 0.88 | > 3.68 |
| **Psychopathy** | 2.4 | 1 | > 3.40 |

**Table 3. Intercorrelations among Short Dark Triad (SD3) sub-scales**

|  | **Machiavellianism** | **Narcissism** | **Psychopathy** |
| --- | --- | --- | --- |
| **Machiavellianism** | **—** | **—** | **—** |
| **Narcissism** | **0.17*** | **—** | **—** |
| **Psychopathy** | **0.31*** | **0.32*** | **—** |

*** *p* < .05 *** *p* < .001**

**Table 4: Brain areas of IC6 – The Central-Executive Network**

| **Region of Interest (ROI)** | **Voxel** | **Peak statistics** | **MNI coordinates** |
| --- | --- | --- | --- |
| 100% of toITG l (Inferior Temporal Gyrus, temporooccipital part Left) | 697 | 40.6 | (-52,-54,-16) |
| 100% of IFG tri l (Inferior Frontal Gyrus, pars triangularis Left) | 650 | 54.2 | (-50,+28,+8) |
| 100% of IFG oper l (Inferior Frontal Gyrus, pars opercularis Left) | 766 | 53.9 | (-50,+14,+16) |
| 98% of toMTG l (Middle Temporal Gyrus, temporooccipital part Left) | 850 | 40.6 | (-58,-52,+0) |
| 93% of AG l (Angular Gyrus Left) | 888 | 31.7 | (-50,-56,+30) |
| 92% of pSMG l (Supramarginal Gyrus, posterior division Left) | 975 | 37.6 | (-54,-46,+34) |
| 91% of Thalamus l | 1240 | 23.3 | (-10,-20,+6) |
| 83% of pTFusC l (Temporal Fusiform Cortex, posterior division Left) | 714 | 16.1 | (-38,-30,-24) |
| 81% of toITG r (Inferior Temporal Gyrus, temporooccipital part Right) | 629 | 23.3 | (+56,-50,-18) |
| 79% of MidFG l (Middle Frontal Gyrus Left) | 2303 | 53.6 | (-40,+16,+42) |
| 72% of Cereb7 r (Cerebelum 7b Right) | 377 | 31.1 | (+30,-72,-48) |
| 71% of FOrb l (Frontal Orbital Cortex Left) | 1197 | 30.1 | (-32,+26,-16) |
| 69% of sLOC l (Lateral Occipital Cortex, superior division Left) | 3411 | 45.7 | (-32,-70,+42) |
| 68% of Cereb2 r (Cerebelum Crus2 Right) | 1448 | 30.5 | (+26,-78,-40) |
| 68% of aTFusC l (Temporal Fusiform Cortex, anterior division Left) | 215 | 11.3 | (-32,-4,-40) |
| 66% of aSMG l (Supramarginal Gyrus, anterior division Left) | 630 | 28.5 | (-54,-34,+40) |
| 64% of Putamen l | 551 | 16.3 | (-26,-4,+2) |
| 64% of Pallidum l | 192 | 13.2 | (-20,-6,+0) |
| 63% of FO l (Frontal Operculum Cortex Left) | 224 | 22.9 | (-40,+22,+4) |
| 60% of pMTG l (Middle Temporal Gyrus, posterior division Left) | 832 | 38.4 | (-60,-34,-8) |

**Table 5: Brain areas for IC15 - the posterior hub of the DMN**

| **Table 4:** **Brain areas for IC15 - the posterior hub of the DMN** | | | |
| --- | --- | --- | --- |
| **Region of Interest (ROI)** | **Voxel** | **Peak statistics** | **MNI coordinates** |
| 100% of Precuneous (Precuneous Cortex) | 5587 | 61.2 | (+0,-60,+38) |
| 100% of Accumbens r | 84 | 12.4 | (+10,+12,-6) |
| 100% of Accumbens l | 107 | 13.4 | (-10,+12,-8) |
| 99% of Thalamus l | 1348 | 29.5 | (-10,-20,+6) |
| 98% of PC (Cingulate Gyrus, posterior division) | 2357 | 49.1 | (+0,-36,+30) |
| 97% of Thalamus r | 1226 | 30.3 | (+10,-18,+6) |
| 96% of AG l (Angular Gyrus Left) | 914 | 29.4 | (-50,-56,+30) |
| 90% of HG r (Heschl's Gyrus Right) | 253 | 8.3 | (+46,-18,+8) |
| 90% of Cereb9 r (Cerebelum 9 Right) | 717 | 14.4 | (+10,-50,-46) |
| 90% of AG r (Angular Gyrus Right) | 1323 | 30 | (+52,-52,+34) |
| 84% of Cereb9 l (Cerebelum 9 Left) | 717 | 16 | (-10,-50,-46) |
| 83% of SPL l (Superior Parietal Lobule Left) | 1219 | 29.9 | (-30,-50,+56) |
| 80% of HG l (Heschl's Gyrus Left) | 248 | 9.9 | (-44,-22,+8) |
| 79% of pMTG l (Middle Temporal Gyrus, posterior division Left) | 1088 | 16.5 | (-60,-30,-8) |
| 70% of pSMG l (Supramarginal Gyrus, posterior division Left) | 744 | 27.1 | (-54,-48,+34) |
| 70% of Pallidum l | 210 | 13.1 | (-18,-6,-2) |
| 69% of SPL r (Superior Parietal Lobule Right) | 1017 | 26.1 | (+32,-48,+56) |
| 67% of AC (Cingulate Gyrus, anterior division) | 1733 | 37.3 | (+0,+24,+22) |
| 66% of SCC l (Supracalcarine Cortex Left) | 48 | 22.8 | (-14,-66,+16) |
| 64% of sLOC l (Lateral Occipital Cortex, superior division Left) | 3159 | 28.5 | (-30,-70,+44) |
| 60% of pMTG r (Middle Temporal Gyrus, posterior division Right) | 818 | 9.9 | (+60,-22,-10) |

**Figure 5. Histograms with Kernel Density Estimate (KDE) plots to visualize the distribution of the data**

**
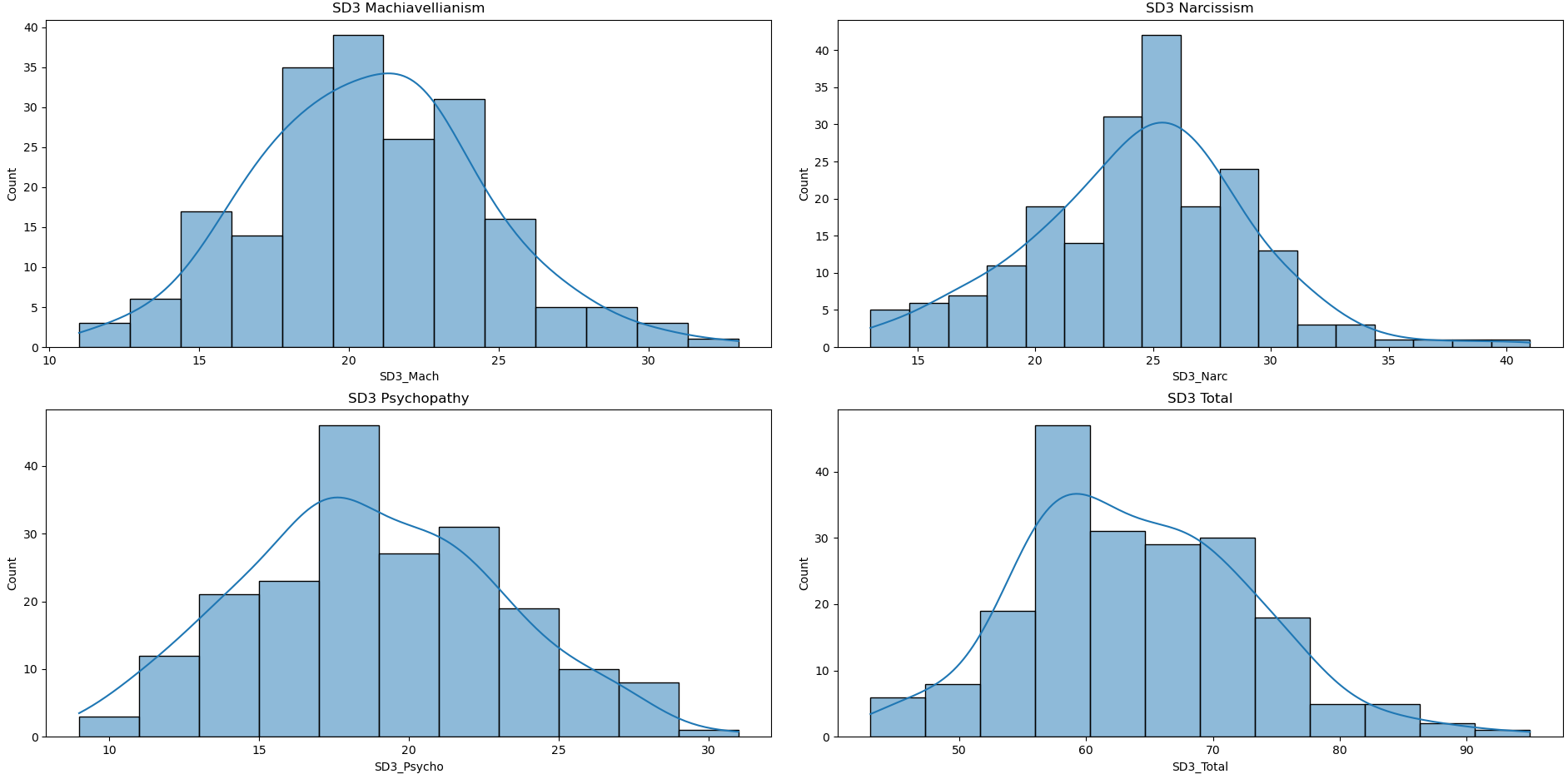
**

**Figure 6. QQ Plots for Assessing Normality of SD3 Dimensions**

**
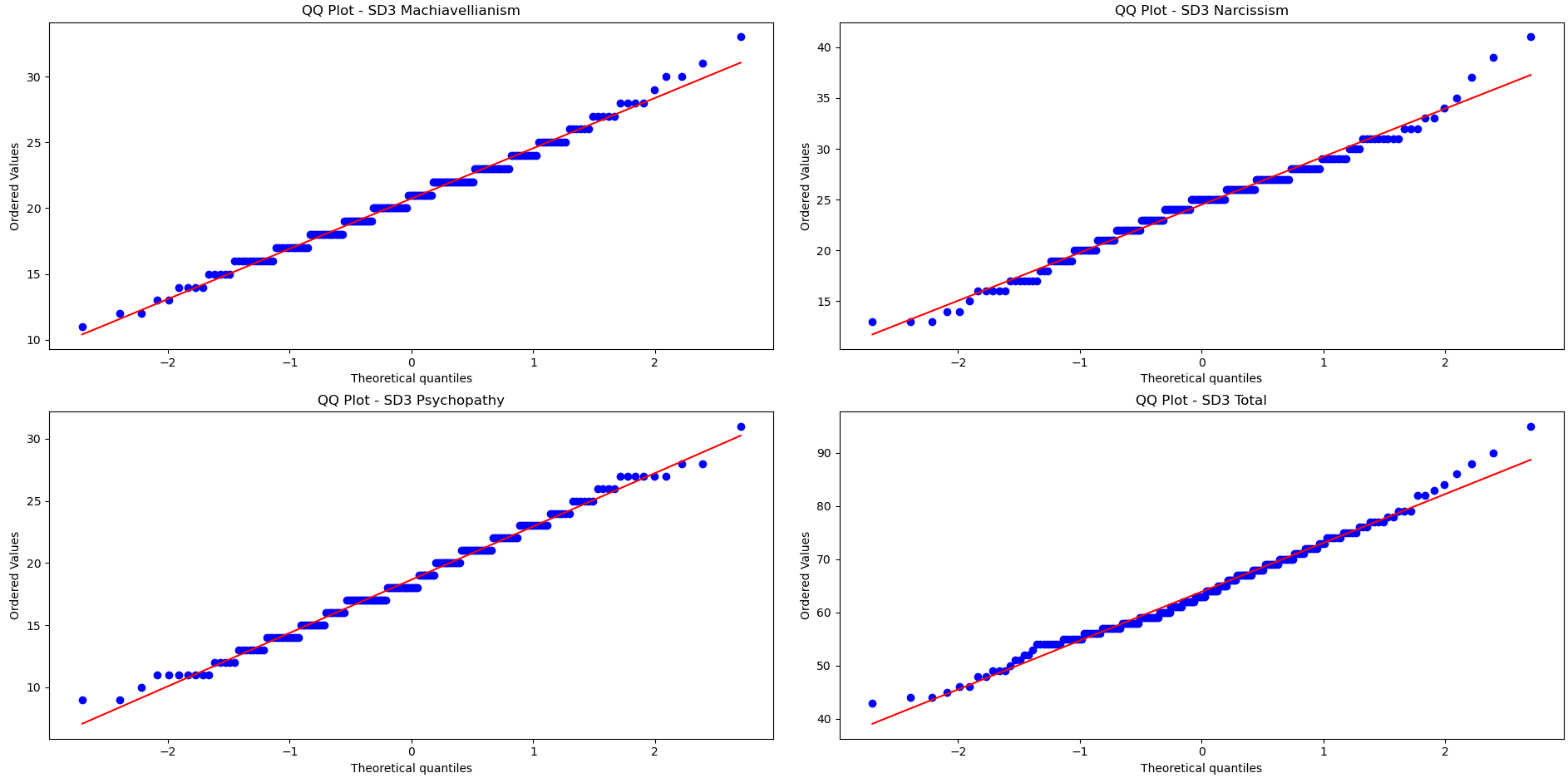
**

We have run distribution tests for both collective and individual components of the Dark Triad by using the Shapiro-Wilk test on JASP (see below Table 7).

**Table 6. Normality Test for Dark Triad Traits**

|  | **SD3_Mach** | **SD3_Narc** | **SD3_Psycho** | **SD3_Total** |
| --- | --- | --- | --- | --- |
| **Shapiro-Wilk** | 0.99 | 0.98 | 0.99 | 0.99 |
| **P-value of Shapiro-Wilk** | 0.18 | 0.02 | 0.14 | 0.069 |

The distribution of the Dark Triad total is normal. The Machiavellianism (SD3_Mach) and psychopathy (SD3_Psycho) dimensions did not significantly deviate from normality (p = 0.18, p = 0.14 respectively). However, the narcissism (SD3_Narc) dimension showed a significant deviation from normality (p = 0.02). For the SD3_Total score, the distribution did not significantly deviate from normality (p = 0.069). To address the non-normality of the dimension of narcissism, we utilized the non-parametric test Spearman’s rank correlation to explore the relationships between Machiavellianism, narcissism, and psychopathy and the 2 neural functional networks detected by gICA (IC6 and IC15). We found that IC6 was positively correlated with Machiavellian traits (*ρ* = 0.162, p < 0.023).
